# Prostaglandins in the Inflamed Central Nervous System: Potential Therapeutic Targets

**DOI:** 10.2174/0113894501323980240815113851

**Published:** 2024-08-22

**Authors:** Chynna-Loren Sheremeta, Sai Yarlagadda, Mark L. Smythe, Peter G. Noakes

**Affiliations:** 1 Institute for Molecular Biosciences, The University of Queensland, St. Lucia, QLD 4072, Australia;; 2 School of Biomedical Sciences, The University of Queensland, St. Lucia, QLD 4072, Australia;; 3 Queensland Brain Institute, The University of Queensland, St. Lucia, QLD 4072, Australia

**Keywords:** Arachidonic acid, neuroinflammation, neurodegeneration, microglia, prostaglandins, NSAIDs, central nervous system disorders

## Abstract

The global burden of neurological disorders is evident, yet there remains limited efficacious therapeutics for their treatment. There is a growing recognition of the role of inflammation in diseases of the central nervous system (CNS); among the numerous inflammatory mediators involved, prostaglandins play a crucial role. Prostaglandins are small lipid mediators derived from arachidonic acid *via* multi-enzymatic pathways. The actions of prostaglandins are varied, with each prostaglandin having a specific role in maintaining homeostasis. In the CNS, prostaglandins can have neuroprotective or neurotoxic properties depending on their specific G-protein receptor. These G-protein receptors have varying subfamilies, tissue distribution, and signal transduction cascades. Further studies into the impact of prostaglandins in CNS-based diseases may contribute to the clarification of their actions, hopefully leading to the development of efficacious therapeutic strategies. This review focuses on the roles played by prostaglandins in neural degeneration, with a focus on Alzheimer’s Disease, Multiple Sclerosis, and Amyotrophic Lateral Sclerosis in both preclinical and clinical settings. We further discuss current prostaglandin-related agonists and antagonists concerning suggestions for their use as future therapeutics.

## 1 INTRODUCTION

**Fig. (1):**
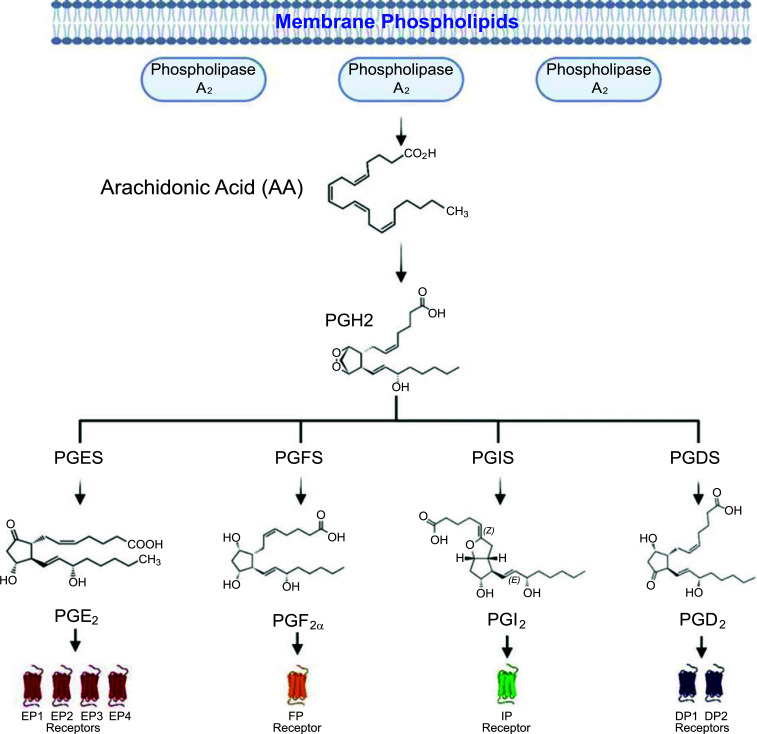
Biosynthetic pathway of prostaglandins and their associated receptors. The arachidonic acid cascade to generate prostaglandins and their downstream receptors. Arachidonic acid is derived from phospholipids, which are released from the plasma membrane by cytosolic phospholipase A2 and is then sequentially metabolised to prostaglandins G2 and H2 (PGH2) *via* cyclooxygenases 1 and 2 (COX-1/2). Specific prostaglandin synthases then metabolise PGH2 to their respective prostaglandins. This includes prostaglandin E synthase (PGES) to prostaglandin E2 (PGE2), prostaglandin D2 synthase (PGDS) to prostaglandin D2 (PGD2), prostaglandin I2 synthase (PGIS) to prostaglandin I2 (PGI2), and prostaglandin F2α synthase (PGFS) to prostaglandin PGF2α (PGF2α). Once synthesised, each prostaglandin can exert its functions through binding to its respective G-protein coupled receptor: PGE2 to EP1-4, PGD2 to DP1-2, PGI2 to IP, and PGF2α to FP. The figure was modified from Yagami *et al.* (2016) [67] and created on Biorender.com.

Prostaglandins (PGs) are small lipid mediators of inflammation that are produced when arachidonic acid is released from membrane phospholipids by cytosolic phospholipase A2α (Fig. **1**). The arachidonic acid can then be oxidised *via* cyclooxygenases 1 and 2 (COX-1/2) to generate prostaglandin G2, followed by reduction to unstable endoperoxide prostaglandin H2 (PGH2), as outlined previously [1]. PGH2 functions as a substrate, allowing for isomerisation by specific synthases to produce its respective prostaglandin, including prostaglandin E2 (PGE2), prostaglandin F2α (PGF2α), prostaglandin I2 (PGI2, also referred to as prostacyclin) and prostaglandin D2 (PGD2; Fig. **1**). Prostaglandins exert their function by activation of specific G-protein coupled receptors (GPCRs) and play crucial roles in maintaining homeostasis of physiological systems and pathophysiological processes [1]. Each PG has its own specific receptor that is comprised of subfamilies, such as the E prostanoid receptors 1-4 (EP 1-4), D prostanoid receptors 1-2 (DP 1-2), F prostanoid receptor (FP) and the prostacyclin receptor (IP), which bind PGE2, PGD2, PGF2α, and PGI2 respectively (Fig. **1**, Table **1**). These receptors have varying tissue distribution and signal transduction pathways, with activation of these receptors leading to different functionality. The role of PGs and their receptors in inflammatory peripheral diseases has been extensively reviewed [2-4] and explored clinically in conditions like Duchenne muscular dystrophy (Clinicaltrials.gov ID: NCT04587908), asthma (Clinicaltrials.gov ID: NCT 02563067), and atopic dermatitis (Clinicaltrials.gov ID: NCT01785602). However, the action of each PG in the central nervous system (CNS) is still not well understood and remains in the preclinical stage of literature. Recently, the role of arachidonic acid metabolites in neurological disorders was discussed [5], however, all upstream metabolites of the arachidonic acid pathway are out of the scope of this review. What follows is a brief description of each prostaglandin and its associated receptors, outlining their involvement in the CNS. Later, we discuss the literature surrounding PGs in a more current disease-specific context, including related therapeutics and future directions. It is evident that the therapeutic space surrounding PGs is rapidly evolving, therefore, it is necessary to explore these treatments for CNS-inflammatory conditions such as Alzheimer's Disease, Multiple Sclerosis, and Amyotrophic Lateral Sclerosis. It is our hope that PG agonism or antagonism will lead to a greater understanding of the biological mechanism of these devasting diseases, thereby reducing inflammation and ameliorating disease symptoms.

## 2 PROSTAGLANDINS AS LIPID MEDIATORS

Prostaglandin E2 is generated from PGH2 *via* three enzymes: the microsomal PGE2 synthases (mPGES-1 and mPGES-2) and cytosolic PGE2 synthase (cPGES). mPGES-1 is an inducible synthase that is expressed by activated microglia [6], suggesting its importance in neuroinflammation. Of all the prostaglandins, PGE2 is the most well-studied in neuroinflammation, and its role has been elucidated in brain diseases especially ischemic injury. PGE2 modulates the expression of inflammatory mediators through microglia by activation of its four receptors (EP1-4), which can be found on glial and neuronal cells throughout the CNS). Although the dual role of PGE2 in the CNS was reviewed previously [7], novel studies have emerged in the past decade. PGE2 has been found to limit cytokine and prostaglandin production through EP2 receptor activation in a model of lipopolysaccharide-induced neuro-inflammation [8]. Its anti-inflammatory effects were similarly observed through the decreased expression of inducible nitric oxide synthase through EP2 activation [9]. The role of the EP2 receptor in CNS and peripheral diseases, as well as its therapeutic potential, has been reviewed [10]. Moreover, studies have investigated the anti-inflammatory role of other PGE2 receptors, like EP4. One study observed that the administration of EP4 agonist significantly improved neurological dysfunction, blood-brain barrier damage, and brain oedema after subarachnoid haemorrhage [11]. An EP4 selective agonist was also observed to decrease an inflammatory response in lipopolysaccharide-induced gene expression in the hippocampus and isolated adult microglia [12]. The role of the EP4 receptor in disease and therapy was reviewed in detail [13, 14]. Conversely, the EP3 receptor demonstrated a role in arachidonic acid-induced inflammation [15]. Evidence also suggests the inflammatory role of EP1 and EP3 activation, which significantly increased IgE-mediated histamine release in mast cells [16]. It appears that the anti- and pro-inflammatory effects of PGE2 in the CNS are receptor-dependent, which will be explained further in a disease-specific context and has been previously assessed [17]. The roles of EP receptors have been reviewed in an inflammatory and therapeutic context [18, 19].

Prostaglandin D2 is a well-known mediator of inflammation, both in the periphery and the CNS. It functions to control vascular permeability, chemotaxis, and antigen presentation, as well as inhibits platelet aggregation and promotes vasodilation and bronchoconstriction [4, 20]. There are two synthases that are responsible for the generation of PGD2: the haematopoietic prostaglandin D2 synthase (HPGDS, also known as the glutathione-dependent haematopoietic PGD synthase) and the lipocalin-type prostaglandin D2 synthase (LPGDS). While HPGDS has predominately enzymatic properties, LPGDS can function as both a synthase and an intracellular transporter. HPGDS is typically found in the periphery as it is responsible for the biosynthesis of PGD2 in antigen-presenting cells [21], mast cells [22], type 2 helper T- lymphocytes [23], and megakaryocytes [24]. Conversely, LPGDS is considered the brain-type PGD2 as it was originally believed to be solely responsible for the production of PGD2 within the CNS and is found to be in elevated levels from lipopolysaccharide-stimulated microglia [25]. However, the presence of HPGDS within the brain has since been confirmed [26]. It has been found that HPGDS is expressed by microglia and that PGD2-induced microglial activation may lead to neuroinflammation [27]. In a healthy individual, PGD2 is the most prevalent PG within the brain [28], functioning as a neuromodulator of various central actions such as the induction of non-rapid eye movement sleep [29] and regulation of nociception26. PGD2 mediates its effects through activation of its two GPCRs, DP1 and DP2 (also referred to as the chemoattractant receptor-homologous molecule expressed on Th2 cells, CRTH2). The DP1 receptor is coupled to Gαs [30], whilst DP2 is coupled to Gαi [31], resulting in divergent effects of cAMP generation and, therefore, downstream effects, such as its inflammatory [32-34] and anti-inflammatory roles [35, 36]. Whilst LPGDS is generally kept to tissue-based expression, HPGDS is localised to the cytosolic aspect of immune and inflammatory cells [37].

As shown in Fig. (**2**), PGD2 can also be non-enzymatically degraded (dehydrated) to generate the J-series of PGs, including 15-Deoxy-Δ12,14-Prostaglandin J2 (15d-PGJ2), PGJ2 and Δ12-PGJ2. Unlike PGD2, 15d-PGJ2 can exert its effect through binding to the peroxisome proliferator-activated receptors gamma (PPARγ), as well as binding to DP2 with a similar affinity as PGD2 [38]. PPARγ agonists have been shown to exert anti-inflammatory actions, acting as negative regulators of monocytes and macrophages and dose-dependently inhibiting the generation of proinflammatory cytokines (*e.g.*, TNF-α, interleukin (IL)-1β, and IL-6) [39, 40].

PGI2, or prostacyclin, is the main PG generated by endothelial cells and has a vital role in vascular homeostasis due to its potent vasodilatory and antithrombotic effects. PGI2 has been implicated in microvascular permeability and ischemia/reperfusion injury and has displayed cardioprotective effects; the function of PGI2 was reviewed by Stitham *et al.* (2011) [41]. PGI2 binds to its GPCR, the prostaglandin I2 receptor (IP), which can be found in numerous tissues such as the kidney, liver, lung, platelet, heart, and aorta [1]. The therapeutic potential of PGI2 has been discussed previously [42-44]. While most studies have explored the function of PGI2 in the periphery, not much is currently known about its neuroinflammatory role, as most studies were conducted decades ago [45-47]. PGI2 is very unstable, thus limiting its utilisation experimentally. The IP receptor is expressed mainly in the neurons, rather than the glia, of the rostral region of the brain, including the hippocampus, cerebral cortex, striatum, and thalamus [45, 48]. PGI2 agonism has been observed to enhance remyelination by promoting the migration of oligodendrocyte precursor cells in mouse models of demyelination within the spinal cord [49]. Conversely, IP receptor antagonists diminished remyelination and motor recovery [49]. Interestingly, levels of rat hippocampal PGI2 increased after ischemia/reperfusion injury, perhaps indicating a physiological mechanism to protect the brain against consequent neuronal damage [50]. Similarly, in models of meningitis (lipopolysaccharide injection), PGI2 administration correlated with a reduction in intracranial pressure, less plasma volume loss, and greater arterial oxygenation [51]. Despite these results, some studies report the detrimental effects of PGI2 within neurological disorders like Alzheimer’s disease [52], as explored later in this review.

PGF2α is arguably the lesser-known prostaglandin, with not much literature surrounding its function within the CNS, as compared to PGD2 and PGE2 [53]. In the periphery, PGF2α is a mediator of inflammation and plays a role in renal and cardiac function, regulation of intraocular pressure, and mammalian reproduction [54]. PGF2α binds to its receptor, the prostaglandin F2α receptor (FP), which can be found in various tissues, including the corpus luteum [55], kidney [56], ocular tissues [57], and ventricular myocytes [58]. Regarding CNS diseases, PGF2α levels were found to be significantly higher in most children with migraines [59]. In mouse models of acute brain injury, it has been demonstrated that significant neurological and anatomical improvements were made after antagonising the PGF2α receptor [60], therefore indicating its possible involvement in brain trauma. Conversely, in models of subarachnoid haemorrhage, PGF2α administration promotes precontraction and vasoconstriction of vessel segments, leading to inflammatory cerebral vasospasm [61].

## 3 NEUROINFLAMMATION AND NEURAL DEGENERATION

Various CNS-related diseases have a presence of sustained immune responses, such as Alzheimer’s Disease (AD) [62], Multiple Sclerosis (MS) [63], and Amyotrophic Lateral Sclerosis (ALS) [64]. A prominent feature of these neurodegenerative diseases is the activation of microglial cells. From ongoing investigations into specific inflammatory mechanisms that are involved in disease causation and progression, we now have a better understanding of inflammation-driven neurodegeneration [65, 66]. We now know that neuroinflammation is a critical factor in most CNS diseases, in which alleviating this inflammation is thought to reduce disease severity and slow disease pathogenesis. This may be done by inhibiting druggable targets, including receptors, ion channels, and enzymes. The following subsections will be devoted to the roles of PGs in the following CNS diseases: AD, MS, and ALS. Although previously touched on by Yagami and colleagues (2016) [67] and Famitareshi and Karimian (2020) [68], this up-to-date review focuses on the evidence of PGs being potential therapeutic targets in the inflamed CNS.

## 4 ALZHEIMER’S DISEASE

### 4.1 Background

Alzheimer’s disease (AD) is one of the most common neurodegenerative disorders, leading to dementia in the elderly. AD is clinically characterised through cognitive impairment and memory loss, which greatly impacts the quality of living within patients. Pathologically, AD can be characterised *via* the deposition of β-amyloid protein (Aβ), intracellular accumulation of tau protein, and neurofibrillary tangles [69, 70]. Aβ proteins are comprised of a 36-42 residue proteolytic product generated through β- and γ-secretase cleavage of the amyloid precursor protein, thus forming amyloid fibrils. Moreover, preceding aggregation of these fibrils, the amyloid precursor protein can form a complex with Zn2+, Fe3+, and Cu2+, thus generating free radicals and leading to neural damage [71]. In healthy neurons, tau proteins function to modulate the stability of the microtubules within axons, pre- and post-synaptic compartments of CNS synapses [72]. However, neuronal death may occur through hyper-phosphorylation of tau, causing self-assembly and insoluble aggregates that block nutrient uptake and synapses [73]. AD is increasingly described as a ‘synaptopathy,’ the damage or loss of synapses that arises due to the disease progression [74]. Synaptopathy is a common trait of dementias and the aging brain with cognitive decline. Consequently, synaptopathy reflects the functional degeneration of specific neuronal circuits leading to neuronal death throughout the brain, as detailed by Goel *et al.* (2022) [75], notably for AD in the basal forebrain, hippocampus, and cortical brain regions [76, 77]. While AD progresses, the cholinergic neurons and synapses are the first affected, leading to further neuronal degeneration and the formation of Aβ deposits, as well as tau tangles [78]. As the majority of AD cases are late and sporadic, it is believed that this disease is caused by multiple factors through environmental exposures, with less than 5% of cases related to familial links or genetic mutations [79, 80]. Although there are numerous hypotheses of the cause of AD, arguably the most held hypothesis is the amyloid cascade hypothesis [81], however, this space is rapidly changing [82]. Despite extensive research into the mechanism responsible for the pathologies of AD, there is yet to be a consensus on the exact cause of the disease. Nonetheless, it is evident that neuroinflammation is a key driver in processing this disease through the chronic activation of microglia and, consequently, the release of proinflammatory factors [83, 84], which was reviewed recently [85].

Within the past decade, many investigations into AD have been focused on sustained inflammatory responses; post-mortem tissues of AD patient brains have demonstrated increased inflammatory responses [86-89]. The inflammatory hypothesis is further supported by epidemiological observations surrounding the use of nonsteroidal anti-inflammatory drugs (NSAIDs) that have been shown to reduce the risk of AD [90]. When chronic inflammation is present within the AD brain, microglia progressively lose their capability to remove Aβ yet sustain the overproduction of proinflammatory cytokines. This contributes to increasing neuroinflammation and consequently increasing Aβ accumulation [91]. The activation of microglia and astrocytes invoke proinflammatory pathways that lead to the release of cytokines, reactive oxygen and nitrogen species, and PGs, thus resulting in degenerative changes in neurons [90]. Increased sustained inflammatory responses further exacerbate Aβ and neurofibrillary tangle pathologies.

Despite AD being the most common neurodegenerative disorder, there remains no effective long-term treatment that alters the pathology of the disease in clinical populations [92]. There has been an observed increase in COX (the key enzymes for PG generation (Fig. **1**)) levels within AD patients [93, 94], as discussed [95-97]. COX-2 is especially upregulated in the brains of AD patients, found mainly in the neurons of the frontal cortex, hippocampus, and thalamus [94, 98, 99]. Various authors have also debated the neuroprotective role of NSAIDs like COX-inhibitors [100-103], indicating that some downstream targets like PGs may play a role in neurodegeneration. Whilst exploring upstream metabolites of the arachidonic acid pathway in detail is out of the scope of this review, it is important to highlight that some investigations have discussed the lack of improvement of AD symptoms after NSAID treatment [104-106]. Although the general consensus is that use of these drugs can protect from the onset of dementia in regards to AD, this treatment is only effective when implemented early in disease progression, over a relatively long period of time [107, 108]. As a result, there is concern regarding the adverse effects associated with chronic NSAID use, particularly selective COX-2 inhibitors such as rofecoxib and celecoxib, in elderly populations [109]. Therefore, the evidence implies that NSAIDs are more of a pharmacological sledgehammer than a silver bullet in the treatment of AD, making it clear that a more efficacious and less toxic treatment is required. For example, targeting specific downstream inflammatory molecules of the arachidonic pathway (Fig. **1**), such as through inhibition or activation of selected PGs. For an in-depth review of eicosanoids in AD, refer to the review by Biringer (2019) [110].

### 4.2 Prostaglandin Targeting Rationale and Experimental Therapeutics

As there is an upregulation of the upstream COX levels, it is unsurprising that there is a further upregulation of various PGs in the AD diseased brain, such as PGD2. In healthy individuals, PGD2 is the most common PG found within the brain, functioning to regulate various physiological processes. Despite this, a recent study by Do *et al.* (2023) [111] evaluated various lipid mediators by liquid chromatography-tandem mass spectrometry of the cerebral spinal fluid (CSF) of AD and mild cognitive impaired patients. They found that PGD2 was lower in mild cognitive impaired patients compared to the subjective cognitive impairment cohort; PGE2 and PGF2α were also decreased in mild cognitive impaired and AD patients within an age-matched cohort. Contrary to these findings, one study observed an increase in PGD2 concentration in AD patients from entorhinal cortex samples [112]. Another study evaluated levels of various PGs in AD patients carrying the apolipoprotein ε3 allele to conclude that there were elevated PGD2 levels when compared to a healthy, age-matched cohort [113]. Similarly, an *in vivo* murine study by Mohri *et al.* (2007) [114] discovered that in Tg2576 (AD-model) mice and human AD patients, there was an increase in HPGDS levels and DP1 receptors, which were overexpressed in microglia and astrocytes within senile plaques. These mRNA levels were further upregulated in Aβ depositions [114]. Overall, the evidence suggests that there may be a discrepancy in PGD2 levels between studies due to disease stage when the samples were collected, or the site of collection (CSF or brain).

As touched on previously, PGD2 can bind to two GPCRs, DP1 and DP2, which are expressed in the hippocampus and cerebral cortex [115], and have opposite effects on cAMP production (Table **1**). Liang and colleagues (2005) [115] explored the use of DP1 agonist, BW245C, on neuronal injury in models of acute excitotoxicity. Overall, BW245C prevented neuronal injury, with its neuroprotective effect disturbed *via* protein kinase A inhibitors. This suggests that PGD2 neuroprotection was mediated by the DP1 receptor, increasing downstream cAMP/protein kinase A pathways. By contrast, neurotoxicity occurs when the DP2 receptor is induced, decreasing cAMP [116]. Like HPGDS, LPGDS has also been implicated within the brains of AD patients and Tg2576 mice; LPGDS was localised in amyloid plaques [117]. LPGDS has chaperone capabilities and can couple to Aβ monomers to prevent aggregation, thus suggesting that inhibition of LPGDS may lead to AD onset and progression [117, 118]. Comparatively, LPGDS has been associated with apoptosis in AD plasma [119]. It is evident that PGD2 plays a role in AD, whether that be neuroprotective or neurodegenerative, depending on the precursor synthase or the binding receptor (Fig. **3**). This is also observed for its metabolites, like 15-PGJ2, as well as other PGs, as described below.

**Fig. (2):**
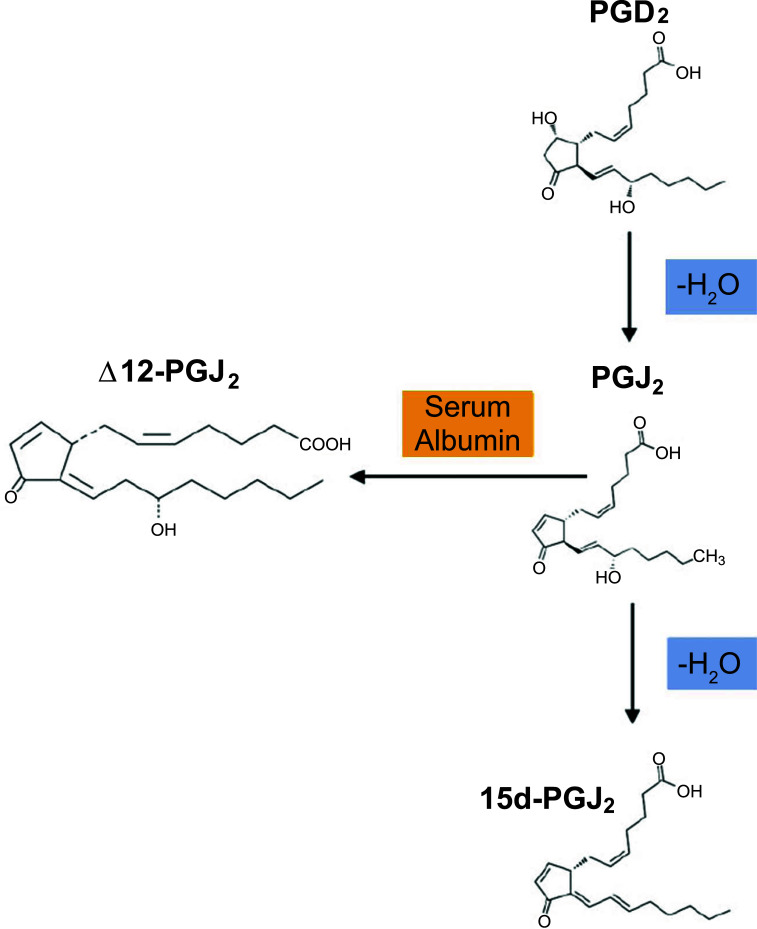
PGD2 is converted into the J-series of prostaglandins. A schematic summarising the generation of J-prostaglandins from PGD2 is shown. The J-series of prostaglandins are derived from prostaglandin D2 (PGD2), which readily undergoes chemical dehydration. This loss in water forms the cyclopentenone prostaglandin J2 (PGJ2). This allows the formation of 15d-PGJ2 through further dehydration as well as Δ12-PGJ2 from serum albumin. Figure created in Biorender.com.

**Fig. (3):**
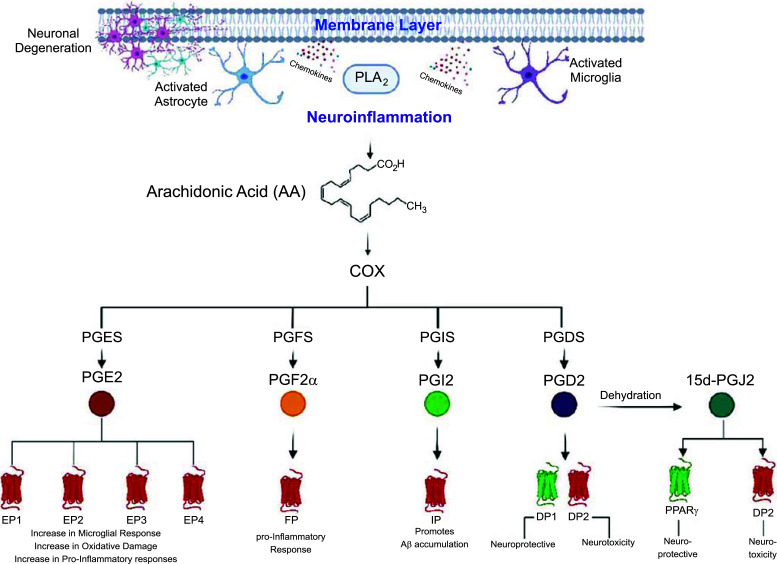
Overview of the roles of prostaglandins in Alzheimer’s Disease. Fig. (**3**) illustrates the involvement of prostaglandins in the pathogenesis and progression of Alzheimer's disease. The process begins with neuroinflammation, marked by the activation of astrocytes and microglia, which release pro-inflammatory chemokines and cytokines. This triggers the release of arachidonic acid (AA) from membrane phospholipids *via* the action of phospholipase A2 (PLA2). Arachidonic acid is then metabolized by cyclooxygenases (COX) into different prostaglandins. PGES is responsible for the isomerisation into PGE2. PGE2 acts on EP receptors (EP1, EP2, EP3, EP4) and is associated with increasing microglial responses, oxidative damage, and proinflammatory responses. PGFS is the enzyme responsible for the production of PGF2α, which acts on FP receptors and contributes to proinflammatory responses. PGIS converts AA precursors into PGI2, which acts on IP receptors and is involved with promoting amyloid-beta (Aβ) accumulation. PGDS converts AA precursors into PGD2, which acts on DP1 and DP2 receptors. DP1 activation is associated with neuroprotective effects, while DP2 activation is linked to neurotoxicity. PGD2 can also be dehydrated into 15d-PGJ2, which acts on PPARγ receptors for neuroprotection and DP2 receptors for neurotoxicity.

15d-PGJ2, which is formed from the dehydration of PGD2, is both a PPARγ and DP2 agonist (Table **1**, Fig. **2**). By utilising this metabolite in microglia culture, Xu *et al.* (2008) [120] demonstrated that 15-PGJ2 inhibits the microglial expression of IL-1β, a pro-inflammatory cytokine that is thought to contribute to AD pathogenesis. 15d-PGJ2 further inhibits microglial generation of other related proinflammatory cytokines such as IL-12 and IL-23 [121]. Together, these findings suggest that the PPARγ agonist may act as a suppressor of microglia activation. However, when bound to the DP2 receptor, 15d-PGJ2 functions as would PGD2, typically leading to neurotoxicity (Fig. **3**) [115].

Elevated PGE2 (Fig. **1**) levels have been found in the CSF of early dementia patients [122]. Ebright *et al.* (2022) [113] also found an increase in PGE2 levels in AD patients carrying the apolipoprotein ε3 allele. The three PGE isoenzymes (cPGES, mPGES-1, and mPGES-2) are also involved in the pathology of AD, and it has since been observed that mPGES-1 is associated with Aβ plaques in the cerebral cortex of human AD patients, as well as Tg2576 mice [123]. Significantly increased levels of mPGES-1 have been found in the neurons, microglia, and endothelial cells of the middle frontal gyrus of those with AD [124]. Whilst an upregulation of mPGES-2 in the pyramidal neurons of the brain has been found from a small study of sporadic and familial AD patients [125], it was suggested by Sluter and colleagues (2023) [126] that most AD-related neuroinflammation is largely mediated by mPGES-1 and EP2 signalling. EP2 receptor activation has been explored in murine AD models. One such study conducted in Aβ inflammation mouse models showed that deletion of EP2 restores regulation of inflammatory responses and Aβ clearance as well as prevented cognitive deficits and loss of synaptic proteins [127]. Similarly, deletion of the EP2 receptor in a familial AD mouse model expressing the Swedish amyloid precursor protein and PS1 mutations (APPSwe-PS1ΔE9 mice) resulted in decreased lipid peroxidation [128]. This decrease in oxidative stress was associated with a significant reduction in levels of Aβ-40 and -42 peptides and amyloid deposition, suggesting that PGE2 signalling through the EP2 receptor has a vital role in the progression of pathology in the APPSwe-PS1ΔE9 model [128]. These findings suggest that EP2 receptor signalling promotes oxidative damage and increased Aβ peptide burden in this AD mouse model, thus providing a rationale for the generation of therapeutics blocking the EP2 receptor in neuroinflammatory diseases like AD (Fig. **3**). Recently, it was found that in Thy1-C/EBPβ transgenic AD model mice, treatment with PGE2 elicited AD-like pathologies [129], possibly due to the binding of more proinflammatory receptors, such as EP2.

PGE2’s other receptors, EP1, 3, and 4 have also been explored in AD models and have been reviewed in the past [130-132]. In brief, evidence shows that in APPSwe-PS1ΔE9 mice, amyloid plaque burden significantly decreases after deletion of EP1 [133]. These mice further exhibited a decrease in proinflammatory gene expression and protein expression when crossed with mice lacking EP3 [134]. When given an EP4 antagonist, AE30208, transgenic mice expressing mutant APP (APP23) displayed an improvement in cognitive performance and decreased levels of Aβ within the brain [135]. Despite signalling through distinct and opposing GPCR pathways (Table **1**), evidence suggests that each receptor contributes to oxidative stress and inflammation in chronic models of AD [132] (Fig. **3**). Interestingly, though, it was reported that EP4 stimulation attenuated microglial inflammatory responses to Aβ42 peptides [136]. However further investigations will be required within *in vivo* models to confirm this finding. Overall, it is evident from the literature that PGE2, its receptors, and isoenzymes can have controversial roles in AD, functioning as both neuroprotective and neurodegenerative; further studies are required to explore these therapeutic targets. For a more detailed review of PGE2 as well as other PGs in AD, refer to Woodling *et al.* (2016) [132], Lima *et al.* (2012) [53], and Fattahi and Mirshafiey (2014) [137].

Regarding PGF2α (Fig. **1**), there are increased levels of its metabolite in AD patients, suggesting that inhibition of PGF2α may be a suitable therapeutic strategy [138, 139]. While some authors have discussed the increase of 8-iso-prostaglandin F2α in dementia and AD [140, 141], the defined role of PGF2α itself remains to be fully elucidated in AD. Similarly, the role of PGI2 (Fig. **1**) in the brain is still poorly understood. In an AD mouse model (APdE9/CP-Tg mice), Womack *et al.* (2022) [52] conducted experiments to evaluate the impact of upregulated PGI2 biosynthesis on these models of AD. They found that increased expression of PGI2 enhanced the advancement of Aβ accumulation and increased the generation of soluble Aβ42 (Fig. **3**). As a result, mice overexpressing both PGI2 and Aβ displayed impaired learning and memory, as well as elevated anxiety-like behaviour. This correlated with the findings of their previous investigation [142]. Likewise, IP-deficient mice demonstrated increased neuronal survival compared to wildtype after ischemic injury [143]. Despite these findings, Ling *et al.* (2020) [144] observed that neurons with higher PGI2 and lower PGE2 showed survival protection and resistance to Aβ-induced neurotoxicity; long-term memory was further restored in AD mice overexpressing PGI2. It is evident that further investigations need to be conducted to confirm the exact role of PGI2 in other transgenic mouse models of AD.

Whilst there have been strides taken to approve various therapeutics for the treatment of AD, many drugs do not progress past phase III trials [145]. This was primarily due to poor accounting for AD subtypes, late therapeutic intervention timing, insufficient biomarkers, and unsatisfactory primary clinical outcome measures surrounding cognitive performance. Current treatments for AD, such as donepezil, galantamine, rivastigmine, and memantine, only display modest benefits and symptomatic treatment [145]. There has yet to be an approved PG antagonist or agonist in the treatment of AD; targeting further downstream of the arachidonic acid pathway (Fig. **1**) will allow for greater precision in the treatment of neuroinflammation within the disease. In support of this strategy, Banik *et al.* (2021) [146] explored the use of an EP2 antagonist in the 5xFAD transgenic mouse model of AD to reveal a reduction in proinflammatory factors, but only in the female mice. Further studies have confirmed that EP2 receptor deletion and antagonism are protective in inflammatory neurodegeneration [147, 148]. Sluter *et al.* (2021) [149] recently discussed the growing space of EP2 antagonism and its application in CNS-based diseases like AD. A phase I clinical trial is currently being conducted (ClinicalTrials.gov ID: NCT05940571), exploring the use of dual EP2/EP4 antagonist, MBF-362, in cancer patients. Similarly, EP2/EP4 antagonist, TPST-1495, has progressed to phase II trials for the treatment of endometrial and colorectal cancers (ClinicalTrials.gov ID: NCT06129604). Preclinical data of these antagonists showed greater efficacy than single EP2 or EP4 antagonists, as discussed [150]. Repurposing these safe, clinically validated therapeutics will be vital for the progression of AD treatment in the future. However, it is recognised that MBF-362 and TPST-1495 are taken orally, which comes with various challenges when treating CNS-based disorders. The inability of drugs to penetrate the blood-brain barrier remains one of the major hurdles of CNS drug development, increasing the translation difficulties from preclinical to clinical therapeutics [151].

Other literature has previously suggested the use of a DP2 antagonist for the treatment of AD. A recent study by Wallace and colleagues (2022) [152] explored the effect of Timapiprant, a DP2 receptor antagonist, in the TgF344-AD transgenic rat model. Timapiprant was originally investigated for atopic dermatitis treatment and progressed to clinical trials (ClinicalTrials.gov ID: NCT02002208) with few adverse effects observed. Wallace *et al.* (2022) [152] showed that AD rats treated with Timapiprant had significantly improved short-term working memory, Aβ plaque load and alleviated neuronal loss and microgliosis. While this is the first study to have investigated this trend, DP2 antagonism appears to be a promising therapeutic target for AD. Downstream of PGD2 and its receptors, APPV717I (AD) mice treated with a PPARγ agonist (pioglitazone, a clinically approved drug for type 2 diabetes) exhibited a reduction in the number of activated microglia and reactive astrocytes in the hippocampus and cortex, consequently decreasing other inflammatory markers like COX-2 and inducible nitric oxide synthase [153]. These mice also showed a significant decrease in total area of Aβ42 deposits in the hippocampus and cortex [153]. Recently, an exploratory phase IIa clinical trial investigated the effects of dual PPARγ/PPARΔ agonist, T3D-959, in subjects with mild to moderate AD; T3D-959 was well-tolerated, and patients exhibited cognitive improvements [154]. Pioglitazone alone was similarly explored for the clinical treatment of AD (ClinicalTrials.gov ID: NCT00982202). The protective effects of the PPARγ agonists in cognitive impairment and neurodegenerative disorders have been reviewed [155, 156]. These findings indicate the need for additional investigations into PPARγ agonists, such as the metabolite of PGD2, 15-PGJ2, for the treatment of AD; a novel series of PPARγ agonists was recently explored in 3xTgAD animals to show attenuated inflammation [157]. However, some authors have discussed the challenges of translating these positive results in animal testing to a clinical setting, especially surrounding the use of PPARγ agonists like pioglitazone [158]. While animal studies have shown the protective effect of pioglitazone, the results of placebo-controlled clinical trials do not reflect similar symptom improvements. This may be due to AD’s complex pathophysiology that fails to translate to one transgenic model, the lack of biological understanding of the disease, and the timing of the therapeutic intervention [158]. Additional preclinical testing must be conducted with tool PG agonists or antagonists to increase our understanding of the inflammatory pathways within an AD population.

## 5 MULTIPLE SCLEROSIS

### 5.1 Background

Multiple sclerosis (MS) is a chronic inflammatory and neurodegenerative disease present within the CNS. It is characterised by relapsing and remitting attacks of inflammation, demyelination, and axonal damage, leading to neurological symptoms and disabilities. Many genetic and environmental factors contribute to the disease, however, the exact aetiology of MS has yet to be elucidated, as touched upon by Dobson and Giovannoni (2019) [159]. MS is characterised through the infiltration of both innate and adaptive immune cells, like macrophages, T, and B lymphocytes, as well as local cells, including microglia and astrocytes, resulting in reactive gliosis [160]. The two main cells that appear to exacerbate tissue damage/lesions are microglia and macrophages, which are commonly discovered post-mortem around MS- damaged axons [161]. There are three clinical forms of MS, the first of which is relapsing-remitting MS, the most common to show initial presentation. As the name suggests, relapsing-remitting MS is defined through relapses or flare-ups in neurological symptoms and lengths of remissions. During relapses, peripheral immune cells infiltrate across the blood-brain barrier; blocking leukocyte tracking from the periphery to the CNS is an effective way of treating relapsing-remitting MS [162]. If left untreated, many relapsing-remitting MS cases will develop into secondary progressive MS, as well as primary progressive MS.

**Fig. (4):**
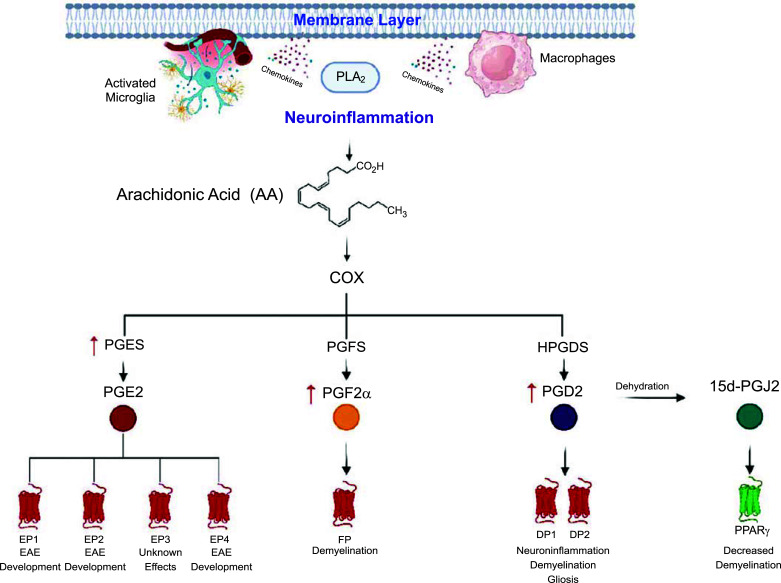
Overview of the roles of prostaglandins in Multiple Sclerosis. Fig. (**4**) illustrates the involvement of various prostaglandins in the neuroinflammatory processes associated with Multiple Sclerosis (MS). The pathway begins with neuroinflammation, initiated by the activation of astrocytes and immune cells (*e.g.* macrophages), leading to the release of PLA2 and the subsequent production of arachidonic acid (AA) from membrane phospholipids. Arachidonic acid is then metabolized by cyclooxygenases (COX) into numerous prostaglandins. PGES converts AA precursors into PGE2. PGE2 acts on EP receptors (EP1, EP2, EP3, EP4) and is associated with increased inflammatory responses, including the activation of immune cells and the promotion of neuroinflammation, exacerbating experimental autoimmune encephalomyelitis (EAE). PGFS is responsible for the isomerisation of PGF2α, which acts on FP receptors and contributes to proinflammatory responses, exacerbating demyelination in MS. PGDS aids in the generation of PGD2, which acts on DP1 and DP2 receptors. DP1 activation is associated with neuroprotective effects, while DP2 activation is linked to neurotoxicity. PGD2 can also be dehydrated into 15d-PGJ2 which activates PPARγ receptors for neuroprotection. The figure was created with Biorender.com.

MS relapse episodes can be treated by glucocorticoids; due to the inflammatory nature of acute stage MS, it is not surprising that glucocorticoids are commonly administered to patients, such as oral prednisone and dexamethasone. However, glucocorticoids are not effective in treating long-term MS and are associated with various adverse effects like gastrointestinal and cardiovascular issues [163]. Current therapeutic options generally encompass disease-modifying treatments, which are categorised into two approaches: continuous immunosuppression and immune system reshaping. The former targets a vast array of immune cell types of the peripheral immune system, such as those of lymphoid (B and T cells) and myeloid lineage (macrophages and dendritic cells). The latter method includes reshaping the immune system into one that is less susceptible to disease activity, feasibly encouraging long-lasting inflammation resolution, as reviewed by Ruiz *et al.* (2019) [162]. This includes the use of chemical therapeutics like alemtuzumab, which ‘resets’ the immune system by depleting NK, T, and B cells, followed by an immune reconstitution. However, clinical use of alemtuzumab is limited due to its adverse effects; expanding levels of repopulated naïve B cells before Tregs can induce systemic loss of immune tolerance and be detrimental to the patient, leading to secondary autoimmunity, infusion-associated reactions, and increased risk of infection [164]. Overall, most available disease-modifying treatments demonstrate favourable effects on relapsing-remitting MS, yet little to no significant benefit in the progressive stages of the disease [165, 166], leading to worsening neurological disability as time passes [167]. This is believed to be due to intrinsic CNS inflammatory and neurodegenerative mechanisms, such as that of axonal degradation and oxidative stress [168]. Despite this, even during the relapsing-remitting stage of the disease, tolerability, efficacy, and safety profiles differ considerably between treatments. For example, some therapeutics may be high in efficacy yet demonstrate a greater risk of serious adverse effects that may prove to be fatal, such as cardiomyopathy (mitoxantrone), bradyarrhythmia (fingolimod therapy), autoimmune thyroiditis (alemtuzumab), as reviewed by Gajofatto and Benedetti (2015) [169]. Current treatment options for MS were recently reviewed by Hauser *et al.* (2020) [170]. It is clear that treatments and associated effects among MS patients are extremely variable, thus leaving an unmistakable gap in standard, efficacious treatment. The increased PG levels in those with MS suggest a function in the immunopathology of the disease and could be a viable druggable target for treatment. Alteration of the arachidonic acid pathway and the upregulation of various PGs in the clinic and models of MS have been reviewed previously [171-175] and summarised below (Fig. **4**) in the context of targeting specific PGs for the treatment of MS.

### 5.2 Prostaglandin Targeting Rationale and Experimental Therapeutics

Most investigations into the role of PGs in MS have been conducted *in vitro* and *in vivo*, using either cell lines or mouse models of MS, as opposed to MS patients. For example, *in vivo* studies can involve the use of the *twitcher* mouse (C57BL/6J-*GALCtwi: GALCtwi/twi*) as in Mohri *et al.* (2006) [34], as well as the experimental autoimmune encephalomyelitis (EAE) mouse, outlined by Robinson and colleagues (2014) [176]. The *twitcher* mouse is a model of human globoid cell leukodystrophy (Krabbe’s disease) that is characterised through demyelination from apoptosis of oligodendrocytes, as recently explored [177]. It is also used as a model of MS since it shares common pathological characteristics, such as microglial infiltration [178] and expression of proinflammatory cytokines like TNF-α [179, 180]. Using brains from the *twitcher* mouse, Mohri and colleagues (2006) [34] revealed that activated microglia showed an upregulation of HPGDS, thus generating a vast amount of PGD2. Hypertrophic astrocytes exhibited an upregulation of the DP1 receptor and expressed the DP2 receptor. Furthermore, Mohri *et al.* (2006) [34] demonstrated that the PGD2- mediated microglia/astrocyte interaction exacerbated neuroinflammation and demyelination through utilising HPGDS-deficient and DP-deficient mice, along with HPGDS inhibitor-treated *twitcher* mice. PGD2 and DP1 binding inhibition led to the suppression of astrogliosis and apoptosis of oligodendrocytes and demyelination. This study overall demonstrated that the blockade of the HPGDS/PGD2/DP1 signalling pathway could be a possible therapeutic pathway for the treatment of MS (Fig. **4**). This was also observed by Zheng and colleagues (2020) [181], where DP1-deficient mice exhibited less demyelination, decreased microglia activation, and reduced leukocyte infiltration in experimental autoimmune encephalomyelitis (EAE) mice (model explained below). Further studies using the *twitcher* mouse have shown that the LPGDS enzyme that aids in the isomerisation of PGD2 from PGH2 is upregulated in oligodendrocytes during the demyelination process. Taniike *et al.* (2002) [182] found that the distribution of LPGDS-positive oligodendrocytes and the severity of demyelination had an inverse relationship, indicating that LPGDS has an anti-apoptotic effect. As demyelination occurs before the apoptosis of oligodendrocytes in the *twitcher* brain, it was suggested that upregulation of LPGDS could suppress apoptosis. Using the double- mutant LPGDS deficient (LPGDS-/-) *twitcher* (GALC*twi/ twi*) mice, the anti-apoptotic role of LPGDS was confirmed [182]. This may be due to the chaperone function of LPGDS, which has been previously explored [117, 118].

Another model of MS is experimental autoimmune encephalomyelitis (EAE). This condition exhibits many key features of MS, including inflammation, demyelination, axonal loss, and gliosis, as discussed by Constantinescu and colleagues (2011) [183]. PGE2 appears to be the PG most associated with EAE onset and progression. Spinal expression of PGE2 was upregulated after EAE induction, with mPGES-1-/- mice demonstrating reduced EAE symptom severity and decreased generation of proinflammatory cytokines than mPGES-1+/+ mice [184, 185]. Receptors EP1/2/4 (Table **1**) were increased in EAE lesions, with immunohistochemistry analyses determining an overt expression of mPGES-1 protein in macrophages and microglia, suggesting that PGE2 may worsen EAE pathology [185]. In support of this idea, EAE mice without EP4 receptor expression (EAE EP4-deficient) mice display a suppression of EAE development, similar to the response after administration of an EP4 antagonist [186]. Various authors have suggested the importance of exploring inhibitors of mPGES-1 [187, 188], particularly in the context of MS treatment [126, 184, 185, 189]. Regarding the potential of other PGs to treat MS in models of EAE, it has been shown that activation of 15d-PGJ2’s receptor PPARγ (Table **1**) by either 15-PGJ2 or Ciglitazone (a selective PPARγ ligand) can inhibit disease progression in EAE mice. This inhibition is characterised by decreased demyelination [190], along with preventing the generation of various proinflammatory molecules such as nitric oxide, cytokines (TNF-α, IL-1β, and IL-6), and the chemokine MCP-1, which are produced from activated microglia and astrocytes [191]. Refer to Fig. (**4**) for an overview of the known effects of each PG in MS.

Whilst these pre-clinical studies promote the role of PGs in MS, limited clinical investigations have been conducted for different stages of the disease. Histological assessments of MS lesions generated by Raine (2017) [192] indicate that activated microglia and macrophages are key players. This can be both advantageous (homeostatic) or unfavourable (over-expression) within a disease such as MS due to the abilities of microglia and macrophages to generate pro- and anti-inflammatory factors like cytokines and PGs. Therefore, it is unsurprising that there is significantly increased COX activity (in secondary progressive MS) [193] and multiple PGs upregulated clinical MS cases. Whilst most PGs evaluations in MS patients were conducted decades ago [194-197], many of these findings agree with current literature. For example, MS patients in active disease generated significantly higher levels of PGD2 and PGE2 from monocytes in their CSF than paired peripheral blood monocytes and monocytes from healthy controls [195]. More recently, the expression of PGE2 from leukocyte cultures was evaluated from human patients of MS who had at least one recent exacerbation, some with chronic progressive or stable MS, as well as healthy controls. It was found that MS patients overall had greater levels of PGE2 compared to the controls, and relapsing and progressive MS patients displayed greater PGE2 and PGD2 levels in blood samples [198]. Similarly, Mattsson *et al.* (2009) [199] found that MS patients demonstrated elevated levels of PGE2 in CSF samples but claimed a lack of correlation with clinical MS scores.

Moreover, the role of other PGs has been briefly explored, such as the expression of the PGF2α receptor, FP (Table **1**). This receptor was found to be bordering the zone of demyelination in active MS lesions in the spinal cord of MS patients [200]. In those with progressive forms of MS, PGF2α was significantly higher in CSF samples compared to other neurological disease controls [201]. LPGDS (formerly known as β-trace) has also been ascribed as the most abundant PG synthase found in the CSF [202]; one study revealed significantly increased LPGDS in the white matter of MS patients [203]. Interestingly, however, there was no observed difference in downstream metabolite, 15d-PGJ2, in plasma levels between healthy controls and patients with different clinical forms of MS [204]. It is difficult to exactly draw conclusions from multiple studies due to a lack of analogous comparisons between disease stage and sample type (*i.e.*, blood or CSF). The limitation of using CSF as a means of measuring PG levels can be made more complex by recent findings that proteins within the CSF are not uniformly distributed in CNS compartments [205]. Therefore, it is evident that future studies need to be conducted to investigate the expression of PGs and their receptors during various stages of MS, to elucidate the most suitable therapeutic.

While there has been progress made in elucidating treatments for MS, effective treatment strategies have yet to be made for progressive stages of the disease. Although there is a wide range of disease-modifying therapies available, there are few guidelines to aid clinicians in selecting suitable, patient-specific treatments based on age, stage, adverse effects and safety profile, efficacy, and tolerability of the therapeutic [206]. Cheap and safer treatments must be explored, such as investigating the benefit of PG receptor agonists/antagonists and their role in inhibiting inflammation within models of MS. There are various anti-inflammatory compounds that have been explored clinically for MS, such as Ibudilast (ClinicalTrials.gov ID: NCT02714036) and HMR1726 (ClinicalTrials.gov ID: NCT01487096). However, there have been limited clinical investigations into the anti-inflammatory effects of targeting downstream of the arachidonic acid pathway. Most literature surrounds preclinical investigations, such as one study that utilised pioglitazone to treat a patient with secondary progressive MS [207]. Pioglitazone, as mentioned previously in this review, is a PPARγ agonist (like 15d-PGJ2, Table **1**) that is FDA-approved to treat type-2 diabetes and can induce apoptosis in activated T-lymphocytes and anti-inflammatory effects in glial cells. After 3 years of daily treatment with the PPARγ agonist, the patient exhibited no adverse effects and showed clinical improvements, thus suggesting further therapeutic testing. Despite being conducted in a limited population, this study still demonstrated the potential for additional investigations into utilising PG receptor antagonists or agonists, such as PPARγ agonists like 15-PGJ2 (Fig. **4**). Moreover, as outlined by literature previously, EP2 receptor antagonists and PGD2 inhibitors prove to be interesting potential therapeutics for the treatment of MS due to their inflammatory nature within MS murine models. The increase of PGD2 and PGE2 is further upregulated in clinical patients, which suggests a disruption in specific PG homeostatic roles in this disease. Interestingly, an investigation previously explored the use of the PGF2α receptor (FP) antagonist, AL-8810, in a cuprizone-induced MS murine model [208]. It was seen that mice treated with this antagonist demonstrated reduced demyelination, glial activation, and inflammatory cytokine expression, as well as an improvement in motor function (Fig. **4**). Whilst there have been no specific PG agonists or antagonists to progress into clinical trials, this space is rapidly changing. As a bridge between preclinical and clinical testing, cerebral organoids generated from human induced pluripotent stem cells (iPSCs) have proven to be useful tools in the drug discovery process. Future drug screening should be focused on implementing similar models, thus allowing successful testing at various disease stages [209]. This may allow the clarification of PGs level fluctuation between patients and demyelination severity; it is predicted that modulating specific PG signalling will have a therapeutic benefit in the treatment of clinical MS.

## 6 AMYOTROPHIC LATERAL SCLEROSIS

### 6.1 Background

Amyotrophic lateral sclerosis (ALS) is a progressive neurodegenerative disease that is characterised by the death of both upper (corticospinal) and lower (alpha) motor neurons, leading to muscle weakness, atrophy, and spasticity [210]. Whilst being somewhat more prevalent in men than women, it impacts approximately 4.42 per 100,000 people, and death typically occurs within 5 years of disease onset due to denervation of respiratory muscles [211, 212]. Although the exact cause of the disease is unknown, as reviewed by Masrori and Van Damme (2020) [210], the chronic inflammatory state is hypothesised to hasten disease progression, resulting in secondary neurotoxicity and the death of motor neurons [213-215]. It is further believed that genetic abnormalities and environmental factors might be at play, increasing disease risk [216]. This is particularly in those with familial ALS, where genes that encode Cu/Zn superoxide dismutase (SOD1) [217] or TAR DNA binding protein 43 (TDP-43) [218] have been mutated. This has given rise to SOD1 gene transgenic mouse models [219], as well as other genetic murine variants [220]. Transgenic mice that carry human motor neuron disease mutated SOD1 (*e.g.*, SOD1G39A), and TDP-43 (*e.g.*, TDP-43Q331K) have pathology akin to those with human ALS, like progressive death of upper and lower motor neurons [221].

Presently, ALS remains to be an incurable disease, with therapeutics aiming to slow disease progression. One of which is the drug riluzole, the most prescribed treatment that aims to block the presynaptic release of glutamate [222]. Nevertheless, there are concerns about its high cost and modest efficacy, and it has been found to only increase median survival by approximately two to three months [223]. Whilst most ALS therapeutics surround slowing disease progression, it must be highlighted that there is an obvious lack of standardised, high-efficacy treatment. Therefore, the apparent role of the arachidonic acid pathway and its downstream metabolites (Fig. **1**) cannot be diminished. Research needs to be conducted to investigate the use of novel PG inhibitors or agonists in treating and slowing the progression of this disease. Arachidonic acid, a precursor of PGs, in elevated levels contributes to motor neuron dysfunction and death in ALS [224]. Whilst many metabolites of the arachidonic acid pathway play a role in ALS, only COX will be briefly discussed, as this review is focused on PGs, its downstream metabolites, and its receptors. The role of COX in ALS was formerly reviewed in detail [225, 226] decades ago and was briefly touched upon by current authors [227-229].

In brief, COX-2 is increased in both SOD1G93A transgenic mice and in ALS-diseased humans [230, 231]. Pompl and colleagues (2003) [232] were one of the first to provide experimental evidence that prophylactic inhibition of COX-2 *via* nimesulide treatment significantly delayed the onset of motor dysfunction within the SOD1G93A transgenic ALS mouse model. This study also saw a greater than two-fold increase in PGE2 content in the spinal cord of the SOD1 mice, compared to wild-type littermates. As expected, nimesulide treatment led to a two-fold decrease in PGE2 content in the spinal cord. More recently, SOD1G93A mice were treated with rofecoxib, a selective COX-2 inhibitor [233]. These mice expressed decreased proinflammatory cytokines after oral COX inhibitor treatment, reduced activation of glial cells, overall postponing disease onset, and modestly prolonging survival. It has been postulated that COX-2 influences ALS through COX-2-derived PGs, promoting inflammatory neurodegeneration *via* induction of glial inflammatory cascades [232]. Despite the apparent benefit of inhibiting COX-2 in treating ALS, there are various adverse effects that are associated with long-term COX-2 inhibition, such as cardiovascular, gastrointestinal, and kidney challenges [234, 235]. Inhibiting upstream of the arachidonic pathway would further downregulate potential beneficial, anti-inflammatory PGs downstream.

### 6.2 Prostaglandin Targeting Rationale and Experimental Therapeutics

**Fig. (5):**
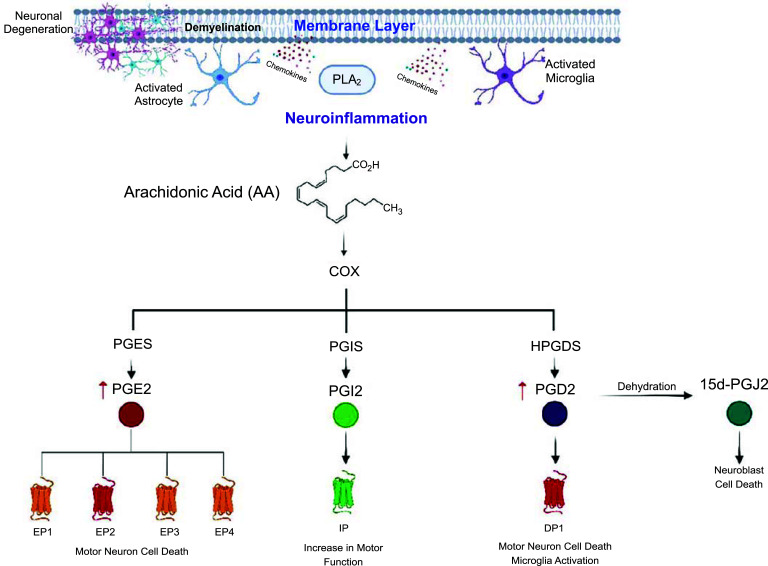
Overview of the roles of prostaglandins in ALS.Fig. (**5**) highlights the roles of various prostaglandins in the neuroinflammatory processes associated with amyotrophic lateral sclerosis (ALS). The pathway begins with neuroinflammation, initiated by the activation of astrocytes and microglia, leading to the release of PLA2 and the subsequent production of arachidonic acid (AA) from membrane phospholipids. Arachidonic acid is then metabolized by cyclooxygenases (COX) into different prostaglandins. PGES is responsible for the production of PGE2. PGE2 activates EP receptors (EP1, EP2, EP3, EP4) and is associated with motor neuron cell death, exacerbating neurodegeneration in ALS. PGIS converts AA precursors into PGI2. PGI2 binds the IP receptor and is involved in increasing motor function, suggesting a potential neuroprotective role in ALS. HPGDS converts AA into PGD2. PGD2 agonises DP1 receptors, leading to motor neuron cell death and microglia activation, which contribute to neuroinflammation and neurodegeneration in ALS. PGD2 can also be dehydrated into 15d-PGJ2, which act on PPARγ receptors and is associated with neuroblast cell death. Figure created with Biorender.com.

Decades ago, studies that focused on specific PGs generated downstream of COX-1/2 (Fig. **1**) proposed that a major mediator of inflammation within ALS is PGE2 [236-238]. This was more recently discussed in a review by Nango *et al.* (2023) [239]. For example, Ilzecka and colleagues (2003) [240] found that PGE2 levels in serum and CSF were significantly higher in ALS patients than that of the control group, leading these investigators to propose that inhibition of PGE2 synthesis may prevent motor neuron death. This conclusion was also drawn by Almer *et al.* (2002) [238] and Liang *et al.* (2008) [213], who found that PGE2-EP2 (Table **1**) signalling participated in the disease pathogenesis of the SOD1G39A ALS model mouse. After EP2 deletion from the SOD1 mice, they observed significantly lower levels of proinflammatory factors such as COX-1 and -2, inducible nitric oxide synthase, and components of the NADPH oxidase complex. These mice further demonstrated improved motor strength and increased survival. More recently, Kosuge *et al.* (2018) [241] found that EP2 was upregulated in the motor neurons of symptomatic ALS mice (B6SJL-Tg [SOD1-G93A]dl 1Gur/); this study showed the importance of EP2 in PGE2-induced cell death in differentiated motor-neuron like cells, as discussed previously [242]. Overall, this study suggested that the selective upregulation of motor neuron EP2 has an important role in PGE2-induced motor neuron death within ALS model mice, not through activation of EP3 receptors (Fig. **5**). These data imply the potential for an EP2 antagonist, such as the novel compound developed by Amaradhi *et al.* (2022) [243]. Administration of this EP2 antagonist successfully displayed a decrease in proinflammatory factors, COX-2, IL-6, and TNF-α, in lipopolysaccharide-activated murine microglia lines. Despite these interesting preclinical results, it is evident that future studies need to be conducted in human primary cell lines before further clinical investigation. Currently, the number of EP2 antagonists is limited, with none yet clinically approved. However, various EP2 antagonists are being developed, with promising preclinical data [148, 243, 244]. One EP2 receptor antagonist (PF-04418948) developed by Pfizer completed a phase I clinical trial (ClinicalTrials.gov ID: NCT01002963). Whilst this compound showed promising results as an orally active, potent, and selective EP2 antagonist, it did not progress further. A more recent study determined that PF-04418948 may not be able to cross the blood-brain-barrier [245]. It is evident that optimisation and development must occur for EP2 antagonists. However, it has been noted that in some instances, activation of specific PGE2 receptors may be neuroprotective, as detailed by Nango and colleagues (2023) [239], this expanding therapeutic space is promising for the treatment of neurodegenerative diseases like ALS.

There is currently limited research surrounding the role of other PGs in human patients and models of ALS. As ALS is characterised by the progressive degeneration and loss of spinal motor neurons, *in vitro* and *in vivo*, studies typically utilise microglia, neuron, and neuroblastoma cell lines, as well as SOD1G93A transgenic mice mentioned previously. The urinary metabolite of PGD2, tetranor PGDM, was found to be significantly higher in ALS populations than in healthy subjects, consequently indicating an increased concentration of PGD2 [246]. Kondo *et al.* (2002) [247] described that 15d-PGJ*2* concentration was accumulated within the motor neurons of the anterior horn in the spinal cord of human sporadic ALS patients, with 15d-PGJ2 inducing cell death in SH-SY5Y neuroblastoma cells (Fig. **5**). This finding is unsurprising as other *in vitro* studies have investigated the effects of upstream PGD2 (Fig. **2**) on ALS-diseased glial cells. One such study indicated that there was increased PGD2 observed in cocultures of ALS glia [248]. Interestingly, PGD2 receptor activation in SOD1 mutated astrocytes leads to motor neuron death [249], with similar evidence suggesting that glia from SOD1G39A mice were toxic to stem cell-derived human motor neurons [250]. This study further indicated that the DP1 receptor plays a vital role in mediating the toxic effects of microglia on motor neurons (Fig. **5**). Genetic ablation of the DP1 receptor in SOD1G39A mice increased life span, diminished microglia activation, and decreased motor neuron death [250]. These findings imply that inhibition of the DP1 receptor may be a viable therapeutic strategy for ALS treatment. A phase II clinical trial (ClinicalTrials.gov ID: NCT01748344) was previously conducted on the novel DP1 antagonist, ONO-4053, showing adequate safety profiles in allergic rhinitis patients [251]. Moreover, a phase II clinical trial was announced in 2021 (ClinicalTrials.gov ID: NCT04705597) for the DP1 antagonist, asapiprant (S-555739) [252]. Whilst these DP1 antagonists are currently aimed at allergic rhinitis and SARS- CoV-2, it is suggested that future studies may explore use in ALS-diseased models. Another therapeutic option may be to target upstream of the receptor, at the synthesis of PGD2. There is a growing space for the use of HPGDS inhibitors for the treatment of inflammatory-based diseases. Currently, there is an HPGDS inhibitor recruiting for phase III clinical trials (ClinicalTrials.gov ID: NCT04587908). This HPGDS inhibitor, TAS-205, was developed by Taiho Pharmaceutical Co., Ltd, aimed for the treatment of Duchenne Muscular Dystrophy. However, the literature suggests a possible therapeutic effect in similar diseases, such as ALS. Another preclinical HPGDS inhibitor was explored in a Duchenne Muscular Dystrophy murine model [253]. This study observed that oral treatment significantly improved grip strength and reduced muscle inflammation and necrosis. Whilst this study focused on the treatment of muscular dystrophy, many of these muscular symptoms are shared with ALS, including increased inflammation, muscle wasting, and weakening [254]. These preclinical data thus indicate the need to conduct additional testing of HPGDS inhibitors in ALS murine models, hopefully ameliorating muscular symptoms that will translate clinically.

Regarding other PG treatments explored, one study by Tada *et al.* (2019) [255] investigated the effect of prostacyclin analogue ONO-1301-MS in SOD1G39A mice. ONO-1301-MS is a novel prostacyclin (PGI2) mimetic that inhibits activity on thromboxane A2 synthase, a prostanoid that is downstream of the arachidonic acid pathway, like PGs. By inhibiting thromboxane, PGH2 (Fig. **2**) can be shunted to induce endogenous PGI2 and PGE2 levels. After subcutaneous administration, the mice displayed significantly improved motor function at 17 weeks of age, as well as increased body weight and motor neuron survival in their spinal cords. However, drug treatment did not extend the average survival time, thus suggesting that ONO-1301-MS treatment increased motor function and ameliorated neurodegeneration during late-stage disease (Fig. **5**) but did not affect overall neuroinflammation. PGI2 and its analogues are currently used in the clinical management of pulmonary arterial hypertension [256]. Due to its already established efficacy and safety profiles, it is further worth exploring its efficacy in the reduction of ALS symptoms. However, despite the ALS field significantly advancing over the past two decades, robust preclinical model systems are still limited. This not only hinders preclinical validation of therapeutics like PG antagonists/agonists but further decreases translatability to a clinical setting. Whilst the hSOD1 mouse is one of the most utilised *in vivo* models of ALS, numerous therapeutics have not successfully translated from this model to human ALS [257, 258]. This may be in part due to the lack of ‘sporadic’ ALS mouse models, unlike the SOD1 mouse, which is more akin to the ‘familial’ disease. Future ALS research needs innovative, complex mouse models to address specific needs in combination with human-based assays such as iPSCs-organoid systems or primary human cell cultures [259]. It is only then that novel, efficacious therapeutics would translate from lab to clinic.

## CONCLUSION

This review discussed the importance of prostaglandins and their associated metabolites in inflammatory CNS-based diseases like Alzheimer’s, Multiple Sclerosis, and Amyotrophic Lateral Sclerosis. We have further outlined the current evidence surrounding antagonising or agonising these mediators and their clinical relevance. PGs play a crucial role in many physiological processes, interacting with multiple receptors to trigger different cellular responses. Elevated PG levels in disease states might correlate with the disease or change during its progression, but this does not prove they cause the pathology. Rather, increased PG levels could be a consequence of other underlying inflammatory processes. It is evident that PGs play dual roles in some diseases, acting in a neuroprotective or neurodegenerative manner. The function of each PG depends on the inflammatory stimulus, phase of the acute response, binding receptor, and other disease mechanisms, which collectively influence whether inflammation resolves or persists.

In developing anti-inflammatory drugs, it is possible that multiple pathways may limit or stop ongoing inflammation. For example, treatment with NSAIDs does show a moderate improvement in many neurodegenerative patients. However, targeting upstream of the arachidonic acid pathway diminishes all mediators downstream. The ideal strategy would involve blocking inflammatory processes while preserving and/or enhancing pro-resolution pathways. Ultimately, for this to be successful, patients must be stratified and treated according to real-time assessments of disease stage, inflammatory status, and causative mechanisms. This requires a deep understanding of the processes driving the inflammatory response to tailor treatments effectively. Targeting specific biochemical pathways that both preserve/enhance pro-resolution and anti-inflammatory effects or using judicious drug combinations is ideal. Despite the structural similarities of PGs possibly presenting challenges in the development of selective drug candidates, there has been success in selectively blocking prostaglandin production at various synthases (*e.g.*, HPGDS, LPGDS, PGES). One of the key limitations is determining if PGs are causative agents or key drivers of a disease, which will require detailed studies that demonstrate direct causal relationships. Utilising selective PG synthases such as these may aid in the elucidation of inflammatory pathways in CNS-based diseases; understanding the underlying biology will be vital in the search for novel therapeutics. Additionally, by selectively inhibiting one PG pathway, we will be able to quantify consequent increase or decrease in neuroprotective factors. It is our hope that the generation and utilisation of these chemical knockouts will allow the validation of therapeutic effects in murine models, in which efficacy will hopefully translate into the clinic.

## KEY POINTS

- Alzheimer’s disease, multiple sclerosis, and amyotrophic lateral sclerosis are neuroinflammatory conditions characterised by altered levels of prostaglandins, which are small lipid mediators of inflammation.
- Prostaglandins can play dual roles in some diseases, acting in a protective or inflammatory manner, depending on the binding receptor.
- Treatments targeting upstream cyclooxygenases (*e.g.* NSAIDs) diminish downstream anti-inflammatory prostaglandins and are associated with various adverse effects with long-term use.
- Inhibiting prostaglandins may aid in diminishing inflammation within the diseased CNS; it is suggested that future research be conducted to synthesise treatments surrounding specific prostaglandin inhibitors and agonists.

## AUTHOR'S CONTRIBUTIONS

It is hereby acknowledged that all authors have accepted responsibility for the manuscript's content and consented to its submission. They have meticulously reviewed all results and unanimously approved the final version of the manuscript.

## Figures and Tables

**Table 1:** Signal transduction of prostaglandin receptors.

**PG**	**Receptor**	**Region of Distribution in CNS**	**GPCR**	**Signalling**
PGD2	DP1	Thalamus, hypothalamus, cortex, hippocampus	Gs	↑cAMP
PGD2 or 15d-PGJ2	DP2	Cortex, hippocampus, thalamus	Gi	↓ cAMP, ↑Ca2+
PGJ-series	PPARγ	Hypothalamus	Nuclear hormone receptor	↓ STAT/JAK
PGE2	EP1	Hypothalamus, thalamus, cortex, hippocampus	Gq	↑IP3/DAG/Ca2+
PGE2	EP2	Cortex, striatum, hippocampus, thalamus	Gs	↑ cAMP
PGE2	EP3	Hypothalamus, thalamus, cortex, hippocampus	Gi	↓ cAMP, ↑Ca2+, Rho
PGE2	EP4	Hypothalamus, thalamus	Gs	↑ cAMP
PGI2	IP	Hippocampus, cerebral cortex, thalamus, striatum	Gs	↑ cAMP
PGF2α	FP	Hippocampus	Gq	↑ IP3/DAG/Ca2+

Each prostaglandin (PG) can bind to its respective receptor, which functions as a G-protein coupled receptor (GPCR): type Gq, Gs and Gi. These receptors are found throughout regions of the central nervous system (CNS), including the thalamus, cortex, hypothalamus, and hippocampus. Once coupled, they exert their function through downstream signalling, either up/downregulating cAMP, calcium, or IP3 (indicated by arrows). The J-series of prostaglandins (PGJ) bind to the PPARγ or DP2 receptor. Depending on the receptor’s signalling, they can have neuroprotectant or neurotoxic properties [2, 137].

## LIST OF ABBREVIATIONS

AD: Alzheimer’s Disease
ALS: Amyotrophic Lateral Sclerosis
Aβ: β-amyloid Protein
CNS: Central Nervous System
COX-1/2: Cyclooxygenases 1 and 2
cPGES: Cytosolic PGE2 Synthase
CSF: Cerebral Spinal Fluid
DP 1-2: D prostanoid Receptors 1-2
EAE: Experimental Autoimmune Encephalomyelitis
EP 1-4: E Prostanoid Receptors 1-4
FP: Prostaglandin F2α Receptor
GPCRs: G-protein Coupled Receptors
HPGDS: Haematopoietic Prostaglandin D2 Synthase
IL: Interleukin
IP: Prostacyclin (Prostaglandin I2) Receptor
IPSCs: Induced Pluripotent Stem Cells
LPGDS: Lipocalin-type Prostaglandin D2 Synthase
mPGES-1: PGE2 Synthase 1
mPGES-2: PGE2 Synthase 2
MS: Multiple Sclerosis
NSAIDs: Nonsteroidal Anti-inflammatory Drugs
PGD2: Prostaglandin D2
PGE2: Prostaglandin E2
PGF2α: Prostaglandin F2α
PGH2: Prostaglandin H2
PGI2: Prostaglandin I2 (also referred to as prostacyclin)
PGs: Prostaglandins
PPARγ: Peroxisome Proliferator-activated Receptors Gamma
SOD1: Cu/Zn Superoxide Dismutase
TDP-43: TAR DNA Binding Protein 43
